# Acute Lymphoblastic Leukemia Arising in* CALR* Mutated Essential Thrombocythemia

**DOI:** 10.1155/2016/6545861

**Published:** 2016-01-21

**Authors:** Stephen E. Langabeer, Karl Haslam, David O'Brien, Johanna Kelly, Claire Andrews, Ciara Ryan, Richard Flavin, Patrick J. Hayden, Christopher L. Bacon

**Affiliations:** ^1^Cancer Molecular Diagnostics, St. James's Hospital, Dublin 8, Ireland; ^2^Department of Haematology, St. James's Hospital, Dublin 8, Ireland; ^3^Department of Clinical Genetics, Our Lady's Children's Hospital, Dublin 12, Ireland; ^4^Department of Histopathology, St. James's Hospital, Dublin 8, Ireland

## Abstract

The development of acute lymphoblastic leukemia in an existing myeloproliferative neoplasm is rare with historical cases unable to differentiate between concomitant malignancies or leukemic transformation. Molecular studies of coexisting* JAK2* V617F-positive myeloproliferative neoplasms and mature B cell malignancies indicate distinct disease entities arising in myeloid and lymphoid committed hematopoietic progenitor cells, respectively. Mutations of* CALR* in essential thrombocythemia appear to be associated with a distinct phenotype and a lower risk of thrombosis yet their impact on disease progression is less well defined. The as yet undescribed scenario of pro-B cell acute lymphoblastic leukemia arising in* CALR* mutated essential thrombocythemia is presented. Intensive treatment for the leukemia allowed for expansion of the original* CALR* mutated clone. Whether* CALR* mutations in myeloproliferative neoplasms predispose to the acquisition of additional malignancies, particularly lymphoproliferative disorders, is not yet known.

## 1. Introduction

Essential thrombocythemia (ET), together with polycythemia vera (PV), and primary myelofibrosis (PMF) are subtypes of the clinically and pathologically related Philadelphia chromosome-negative myeloproliferative neoplasms (MPN). ET is characterized by a sustained peripheral blood thrombocytosis and elevated numbers of mature, large megakaryocytes in the bone marrow and clinically by episodes of hemorrhage and/or thrombosis [1]. Identification of an acquired, clonal mutation has become a fundamental aspect of ET diagnosis [2] with identification of mutations of* JAK2*,* MPL*, and* CALR* likely to be incorporated into the revised World Health Organization diagnostic criteria for this MPN [3].

Although a relatively indolent disorder, there exists a potential for ET patients to transform to a myelofibrotic or leukemic phase. While most leukemic transformations in MPN resemble acute myeloid leukemia [4], transformations to, or coexistence of, an acute lymphoblastic leukemia (ALL) of either B cell or T cell phenotype are uncommon but have been documented in PV [5–10], ET [11–14], PMF [15–17], post-PV MF [18], and MPN unclassified [19] which usually become apparent following treatment with alkylating agents or radioactive phosphorus. Whether all these cases represent a true clonal evolution of the MPN or are the manifestation of two separate diseases remains unresolved in most historical instances. Clinical management of such cases is complex requiring consideration of both pathologies.

From the initial report of* CALR* mutations in MPN, it has become increasingly evident that* CALR*-positive ET patients possess a different phenotype from their* JAK2* V617F-positive counterparts with a younger age, male predominance, higher platelet count, lower hemoglobin, lower leucocyte count, and, perhaps most clinically relevant, a lower risk of thrombosis [20–25]. However, the impact of* CALR* mutations on myelofibrotic and leukemic transformation is less well defined [21–23]. Furthermore, an increased risk of lymphoid neoplasms, particularly mature B cell malignancies such as chronic lymphocytic leukemia (CLL) and non-Hodgkin lymphoma, has been reported in MPN patients compared to the general population [26, 27] suggesting a pathophysiological link between the two diseases. A specific genetic cause or microenvironmental, inflammatory interaction for this predisposition has yet to be identified. A case is described of a hitherto unreported development of ALL in a patient with* CALR* mutated ET.

## 2. Case Report

**Figure 1:**
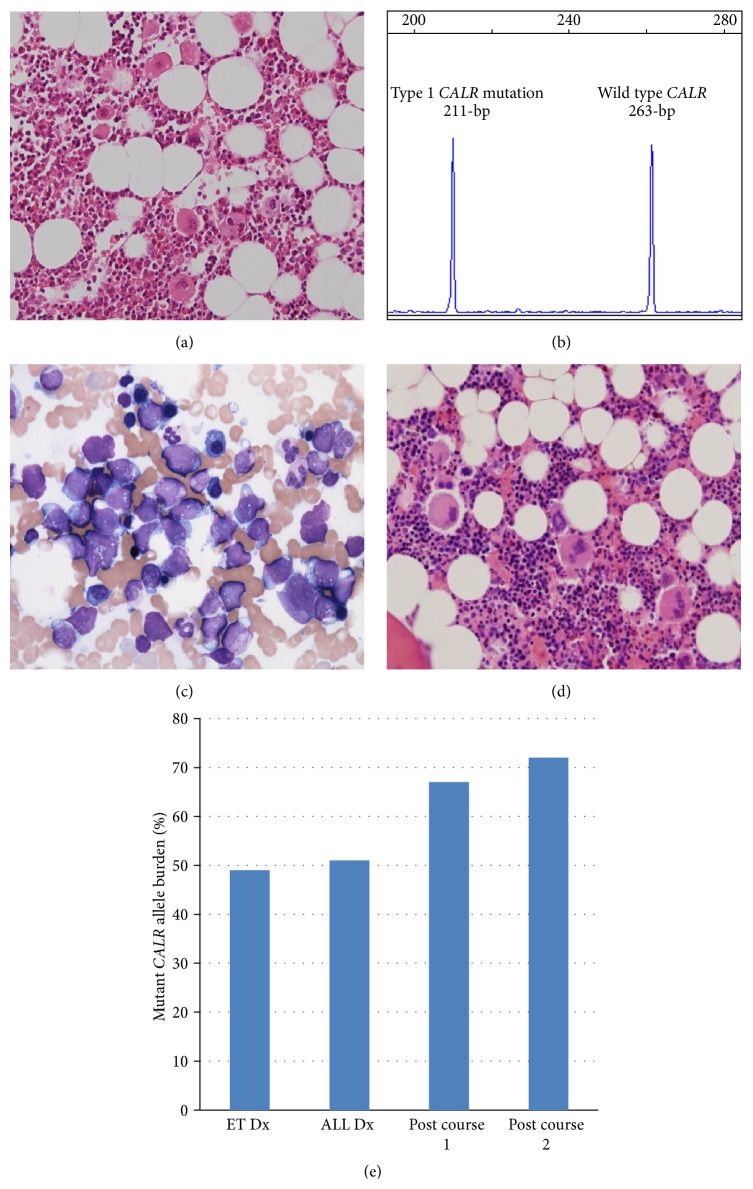
(a) Bone marrow trephine biopsy at diagnosis of essential thrombocythemia (Hematoxylin and Eosin; magnification ×20); (b) fragment length detection of the* CALR* mutation; (c) bone marrow aspirate at diagnosis of acute lymphoblastic leukemia; (d) bone marrow trephine biopsy following two courses of chemotherapy (Hematoxylin and Eosin; magnification ×20); (e) mutant* CALR* allele burdens.

A 65-year-old female presented with an incidentally detected thrombocytosis in 2012. Initial hematological investigation showed a hemoglobin of 13.5 g/dL, white cell count of 6.2 × 10^9^/L, and platelets of 674 × 10^9^/L with platelet anisocytosis and giant platelets on the peripheral blood (PB) film. The bone marrow (BM) aspirate and biopsy were both slightly hypercellular with increased numbers of clustering megakaryocytes (Figure 1(a)) and no increase in reticulin fibrosis consistent with a diagnosis of ET. Cytogenetic analysis was not performed at this time. The* JAK2* V617F mutation was not detected by allele-specific PCR [28]. The patient was commenced on aspirin and hydroxyurea to bring the platelet count within normal range. Retrospective fragment length analysis for* CALR* mutations [29] on the archived, diagnostic, unselected, and PB DNA demonstrated the presence of a type 1 mutation (52-bp deletion, p.L367fs^*∗*^46) at an allele burden of 49% (Figure 1(b)).

Three and a half years after ET diagnosis, the patient presented with left hip pain, hemoglobin of 12.3 g/dL, white cell count of 5.3 × 10^9^/L, platelets of 261 × 10^9^/L, and a lactate dehydrogenase of 1186 IU/L. Macrocytosis, nucleated red blood cells, myelocytes, and 2% blasts of lymphoid appearance were observed on the PB film. In the BM aspirate, blasts with a high N/C ratio with prominent cytoplasmic vacuolation and blebbing accounted for 53% of nucleated cells morphologically (Figure 1(c)) and were CD34+, CD19+, HLA DR+, CD20+, CD38+, TdT+, and CD10− by multicolor flow cytometry and CD34+, PAX5+, TdT+, CD10−, and MPO− by immunohistochemistry, consistent with a diagnosis of pro-B ALL. There was no evidence of increased reticulin fibrosis. Cytogenetic analysis demonstrated a near-triploid clone of 60~67, XXX, +X, +1, −3, −7, +8, −9, add(14)(p11), −15, −16, −17, +18, +18, +19, −20, and +21 [cp7]. The unselected BM* CALR* mutant allele burden at the time of ALL diagnosis was 51%. The patient commenced induction chemotherapy with dexamethasone, idarubicin, vincristine, and methotrexate attaining a morphological, cytogenetic, and immunophenotypic remission. Recovery from induction was associated with a rising platelet count peaking at 1163 × 10^9^/L and an unselected BM* CALR* mutant allele burden of 67%. Following a course of consolidation chemotherapy the patient remained in morphological, cytogenetic, and immunophenotypic remission, with increased megakaryocytes still evident on the BM biopsy (Figure 1(d)). The recovery platelet count peaked at 470 × 10^9^/L with an unselected BM* CALR* mutant allele burden of 72% (Figure 1(e)). The patient was unsuitable for allogeneic hematopoietic stem cell transplantation (AHSCT) and she remains on treatment for both ALL and ET.

## 3. Discussion

The development of ALL in patients with an existing MPN, whether concomitant or representing a true leukemic evolution, has been sporadically documented [5–19]. In the pre-*JAK2* V617F era, only cytogenetic evidence was available to support the existence of distinct entities [30]. However, cell sorting and mutational analysis of hematopoietic stem and progenitor cells in ET and PV cases with coexisting CLL have demonstrated the absence of the* JAK2* V617F and, in one previous case, a* CALR* mutation, in the lymphoid compartment [31–34]. Similar investigations in cases of ALL arising in MPN have distinguished separate clonal origins of the two hematopoietic malignancies [35, 36] therefore weakening the argument for lymphoblastic transformation. However, it remains possible that the ALL could be derived from a malignant subclone prior to the acquisition of the* CALR* mutation. Low numbers of circulating peripheral blood lymphoblasts prohibited such a selection procedure in this case.

Recent evidence suggests that the two most common* CALR* mutation types might influence the MPN phenotype and disease course: type 1 mutations appear to be associated with PMF phenotype whereas type 2 mutations (a 5-bp insertion, p.K385fs^*∗*^47) are more commonly observed in ET [37–39]. Type 1* CALR* mutations in ET, as evident in the patient described herein, are also associated with a higher risk of myelofibrotic transformation although the biological mechanism(s) for this have yet to be clarified: impaired calcium signalling and calcium binding activity to mutant CALR protein have been preferentially demonstrated in type 1* CALR* mutated MPN [39]. At present there is no evidence of equivalence of mutated* CALR* allele burden between peripheral blood and bone marrow; however, of particular note is the mutated* CALR* allele burden after ALL treatment. Mutant* CALR* allele burdens greater than 50% are rare in ET and PMF at diagnosis suggesting the majority of mutations are heterozygous. The relatively high mutant* CALR* allele burdens after ALL treatment might represent loss of heterozygosity in at least some mutant clones, analogous to that observed in the progression of* JAK2* V617F-positive PV [40].

Hydroxyurea is commonly used for the long term treatment of MPN and is well tolerated in the majority of patients. Some early debate suggested that hydroxyurea might contribute to or hastens leukemic transformation; however it was unsure whether transformation was part of the natural history in such instances [41]. Several large studies have subsequently confirmed that hydroxyurea is not associated with myelofibrotic or leukemic transformation in MPN [42–45]. Some studies have reported an increased risk of other cancers, including hematological malignancies, in MPN with myelosuppressive treatment speculated to be a potential cause. Epidemiological evidence of this increased cancer incidence is supported by noting malignancies prior to MPN diagnosis thus eliminating the influence of MPN related treatment [46]. The cooccurrence of MPN and solid tumors may be attributed to the presence of a predisposing* TERT* polymorphism [47]. Another possible common denominator in MPN occurring in conjunction with other malignancies is chronic inflammation, able to potentiate each other's existence and progression [48].

While the coexistence of MPN and mature B cell lymphoproliferative disorders has been documented, cases of MPN and ALL are extremely scarce. We describe, to the best of our knowledge, the clinical course of the first case of pro-B ALL arising in a patient with* CALR* mutated ET. The persistence of the* CALR* mutated clone following treatment for ALL suggests that long term eradication of both diseases would be only achievable with AHSCT.
